# From Anaemia to Arteritis: Unravelling the Diagnosis of Large Vessel Vasculitis

**DOI:** 10.7759/cureus.90647

**Published:** 2025-08-21

**Authors:** Bristee Bose, Mehul Chawla, Sujan Kumar Dey

**Affiliations:** 1 Acute Medicine, University Hospitals Plymouth NHS Trust, Plymouth, GBR

**Keywords:** anaemia, aortic involvement, arm claudication, ct imaging, giant cell arteritis, iron deficiency, large vessel vasculitis, polymyalgia rheumatica, subclavian artery occlusion, vascular inflammation

## Abstract

Large vessel vasculitis (LVV) is a rare but serious inflammatory condition that often presents with nonspecific systemic symptoms, making early diagnosis challenging. This case report describes a 59-year-old female who presented with progressive fatigue, myalgia, weight loss, and iron deficiency anaemia (IDA). The diagnosis of LVV was established following a CT scan performed during the evaluation of IDA, revealing a rare but important cause of her symptoms. Clinical suspicion for vasculitis was initially low despite systemic features. The patient was treated with high-dose corticosteroids followed by disease-modifying antirheumatic drugs, resulting in marked symptomatic improvement and normalisation of inflammatory markers. This case emphasises the importance of considering LVV in the differential diagnosis of patients with anaemia of unclear aetiology and systemic symptoms, especially when vascular involvement is suspected.

## Introduction

Large vessel vasculitis (LVV) is a chronic autoimmune inflammatory condition predominantly involving the aorta and its major branches. It encompasses two major clinical entities: giant cell arteritis (GCA), which primarily affects the cranial arteries in older adults, and Takayasu arteritis (TA), which typically involves the aorta and its proximal branches in young women [1]. The aorta may be affected along its entire course, with the subclavian and common carotid arteries most frequently involved.

Clinically, patients with LVV often present with systemic inflammatory symptoms such as fatigue, fever, weight loss, and arthralgia. Vascular features include limb claudication, absent or diminished pulses, blood pressure discrepancies between limbs, and occasionally visual disturbances [2,3]. These symptoms guide clinicians to suspect LVV and differentiate it from other systemic inflammatory disorders. In GCA, headaches, jaw claudication, and visual symptoms are common, while TA usually presents with limb ischaemia and vascular bruits. Early symptoms are often nonspecific, which delays diagnosis until vascular involvement becomes apparent.

The pathophysiology of GCA involves T-cell activation, macrophage infiltration, and cytokine-mediated vascular inflammation, although the mechanisms remain incompletely understood. TA is characterised by panarteritis involving all layers of the arterial wall, leading to chronic inflammation and fibrosis affecting primarily the media and adventitia [4]. The incidence of GCA varies worldwide, with the highest rates reported in Northern Europe, up to 44 cases per 100,000 individuals aged fifty years or older, and much lower rates in Southern Asia. TA has an estimated annual incidence of one to two cases per million in Japan and 0.4-3.4 cases per million in Europe [5].

Anaemia is a common but often overlooked feature of LVV. It is typically caused by anaemia of chronic disease (ACD), which results from inflammation that disrupts iron metabolism. Iron deficiency anaemia (IDA) can also occur, secondary to gastrointestinal (GI) malabsorption, subtle blood loss, or the effects of inflammatory cytokines. Studies show that cytokines such as interleukin-6 (IL-6), tumour necrosis factor alpha (TNF-α), and interferon gamma (IFN-γ) promote hepcidin production, thereby reducing iron availability. A reported case of idiopathic aortitis demonstrated improvement only after combined iron supplementation and corticosteroid treatment, indicating that both ACD and IDA may contribute [6,7].

This case highlights a diagnostically challenging and rare presentation of LVV in a 59-year-old woman, where typical systemic symptoms such as fatigue, weight loss, and anaemia were initially attributed to GI causes. The delayed diagnosis was ultimately made following computed tomography of the thorax, abdomen, and pelvis (CT TAP) performed during the investigation of her anaemia.

## Case presentation

A 59-year-old woman with a significant smoking history and a family history suggestive of an unspecified aortic pathology presented with progressive fatigue, generalised myalgia, and an unintentional weight loss of 20 kg over three months. These constitutional symptoms (i.e., fatigue, weight loss, and myalgia) were the earliest manifestations of her underlying vasculitis. Initial blood tests revealed IDA, characterised by a low mean corpuscular volume (MCV), a finding that in older adults without an obvious GI source should prompt consideration of systemic causes, especially when accompanied by elevated inflammatory markers (Table 1). Five months before hospital admission, she had been diagnosed with polymyalgia rheumatica (PMR) but declined corticosteroid treatment owing to concerns about side effects.

**Table 1:** Trends in haematologic, inflammatory, and biochemical parameters throughout hospitalization and treatment. Hb: haemoglobin; MCV: mean corpuscular volume; eGFR: estimated glomerular filtration rate; CRP: C-reactive protein; WBC: white blood cell; IU: international unit A hyphen (“-”) indicates that the test was not performed during the hospitalization. Initial iron studies were conducted before admission; subsequent values were obtained after the completion of treatment.

Test Name	Initial Result (on Admission)	Inpatient Result	Subsequent Result (After Treatment)	Units	Reference Range
Hb	98	104	121	g/L	120-155
MCV	76.9	75.2	90.1	fL	80-105
Haematocrit	0.308	0.320	0.369	L/L	(0.37-0.46)
CRP	98	68	2	mg/L	0-15
WBC	9.0	7.4	7.7	×10⁹/L	3.6-9.2
Neutrophils	6.3	5.0	5.0	×10⁹/L	1.7-6.2
Sodium	138	137	139	mmol/L	133-146
Potassium	4.3	4.1	4.2	mmol/L	3.5-5.3
eGFR	>90	>90	>90	mL/min/1.73m²	>90
Alkaline Phosphatase	135	144	59	IU/L	30-130
Alanine Transaminase	10	11	26	IU/L	1-55
Bilirubin	4	3	4	µmol/L	1-20
Serum Iron	8	-	13	µmol/L	9-30
Serum Ferritin	169	-	101	µg/L	30-200
Transferrin	2.70	-	2.87	g/L	1.80-3.82
Transferrin Saturation	11.20%	-	17.61%	%	16-40

Three months before admission, the patient travelled to Egypt, where she experienced a self-limiting upper respiratory tract infection and an episode of chronic diarrhoea, both of which resolved spontaneously. Two months later, she developed worsening fatigue, myalgia, and reduced exercise tolerance, followed by arm claudication symptoms, particularly during activities such as washing dishes, and jaw claudication. She denied fever, chest pain, shortness of breath, rash, joint swelling, or overt GI bleeding. There was no history of melaena, haematochezia, or haematemesis. A quantitative faecal immunochemical test (qFIT) returned borderline positive at 10 µg/g, thought possibly related to the preceding diarrhoea; a repeat qFIT was planned. Upper and lower GI endoscopies were initially scheduled by the gastroenterology team but were cancelled after a contrast-enhanced CT TAP revealed features consistent with LVV, shifting the diagnostic focus away from a GI source. Based on these CT findings, the patient was referred by the radiology team to same-day emergency care and was subsequently admitted to the acute medical unit for further management.

**Figure 1:**
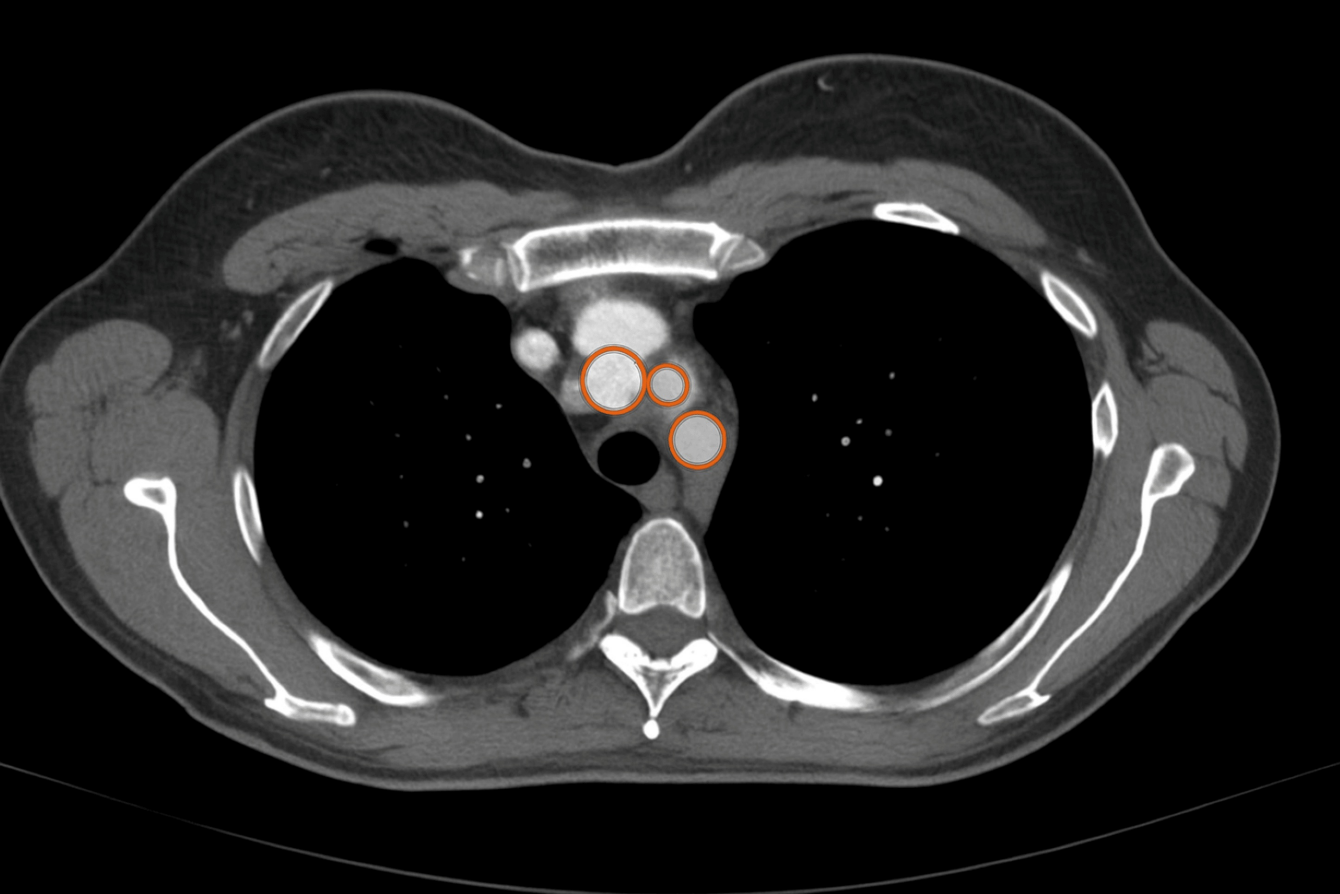
Axial contrast-enhanced CT chest demonstrating diffuse concentric mural thickening of the thoracic aorta (circled in red), most prominent in the ascending and descending segments. These findings are highly suggestive of inflammatory aortitis, consistent with large vessel vasculitis (LVV). This imaging played a pivotal role in prompting further vascular evaluation.

**Figure 2:**
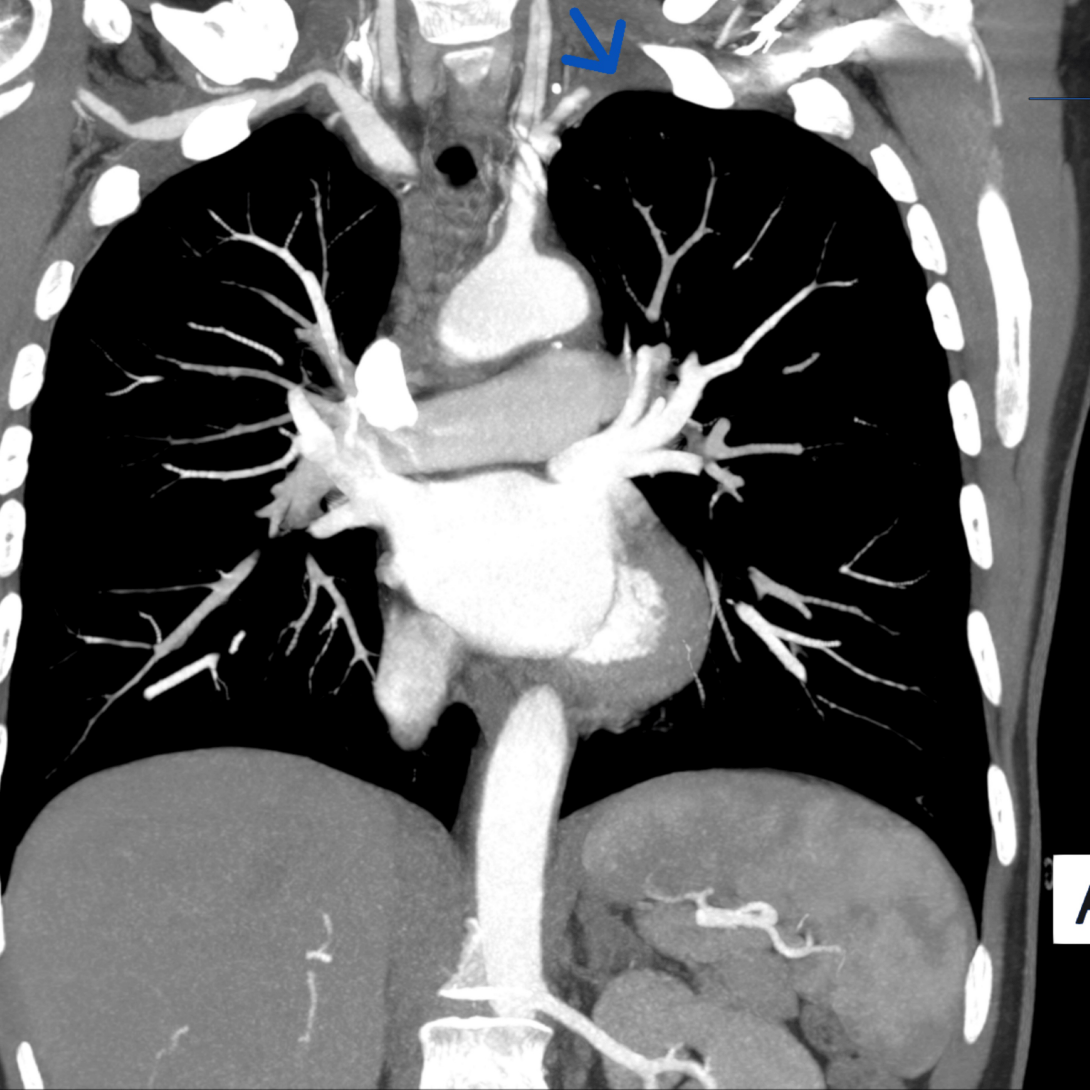
Contrast-enhanced CT TAP showing complete occlusion of the left subclavian artery (blue arrow) approximately 5 cm distal to its origin from the aortic arch. The absence of distal contrast opacification confirms vascular occlusion, a hallmark feature of LVV in this patient, and explains the clinical symptom of left arm claudication. CT TAP: CT of the thorax, abdomen, and pelvis

Contrast-enhanced CT TAP demonstrated diffuse concentric mural thickening of the thoracic aorta (Figure 1) and abdominal aorta, involving the aortic arch and its major branches. Key findings included complete occlusion of the left subclavian artery approximately 5 cm from its origin (Figure 2), proximal occlusion of the right brachial artery secondary to subclavian origin stenosis, and severe stenosis at the origin of the right vertebral artery. Mural thickening extended into the abdominal aorta and common iliac arteries, with mild involvement of the femoral arteries. The coeliac axis and superior mesenteric artery showed mild ostial thickening but no occlusion. The renal arteries were unaffected.

**Figure 3:**
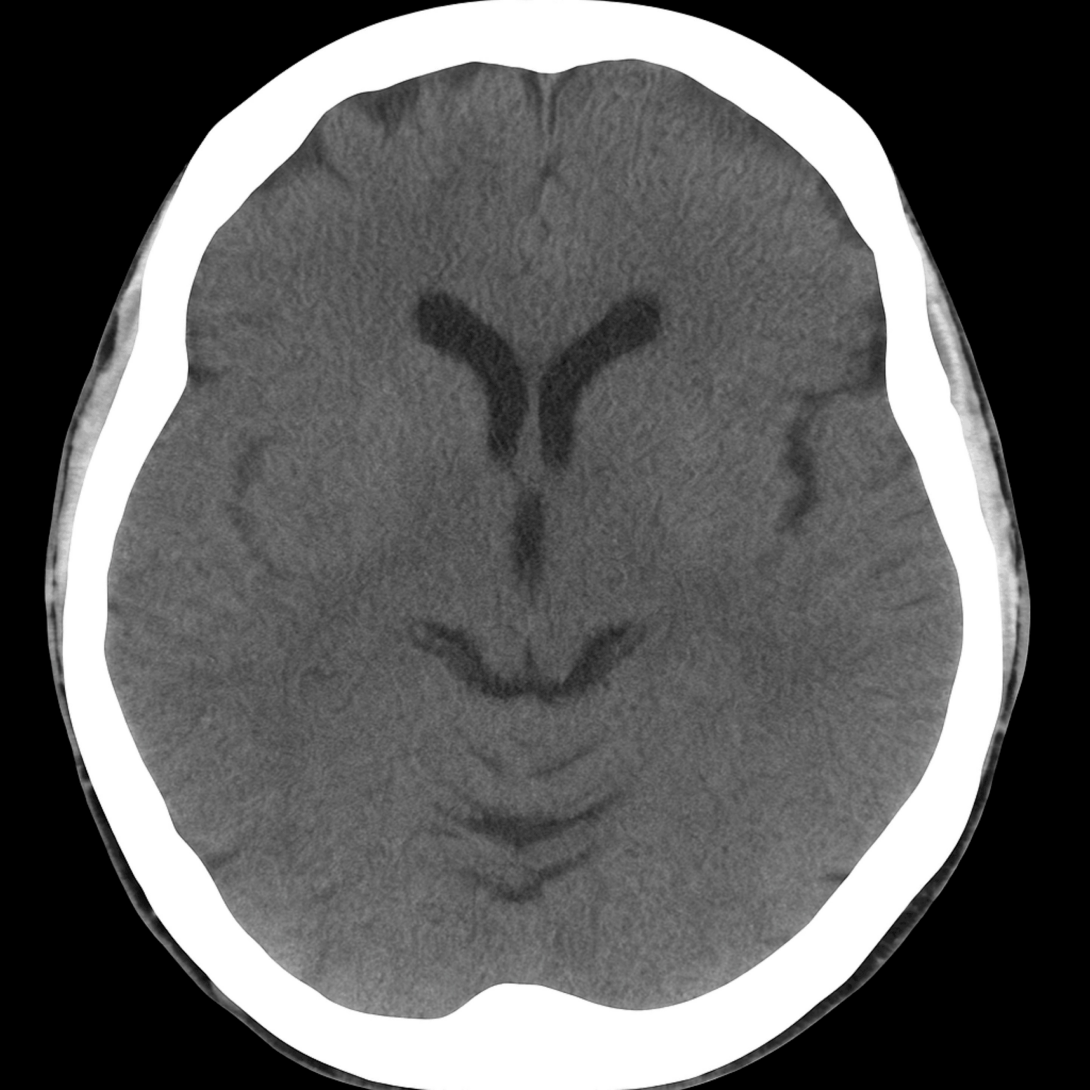
Non-contrast CT head demonstrating no evidence of acute intracranial pathology: no haemorrhage, infarction, mass effect, or ischaemic changes are seen. This imaging was performed to exclude neurovascular complications such as stroke or intracranial vasculitis, which can occasionally accompany LVV.

**Figure 4:**
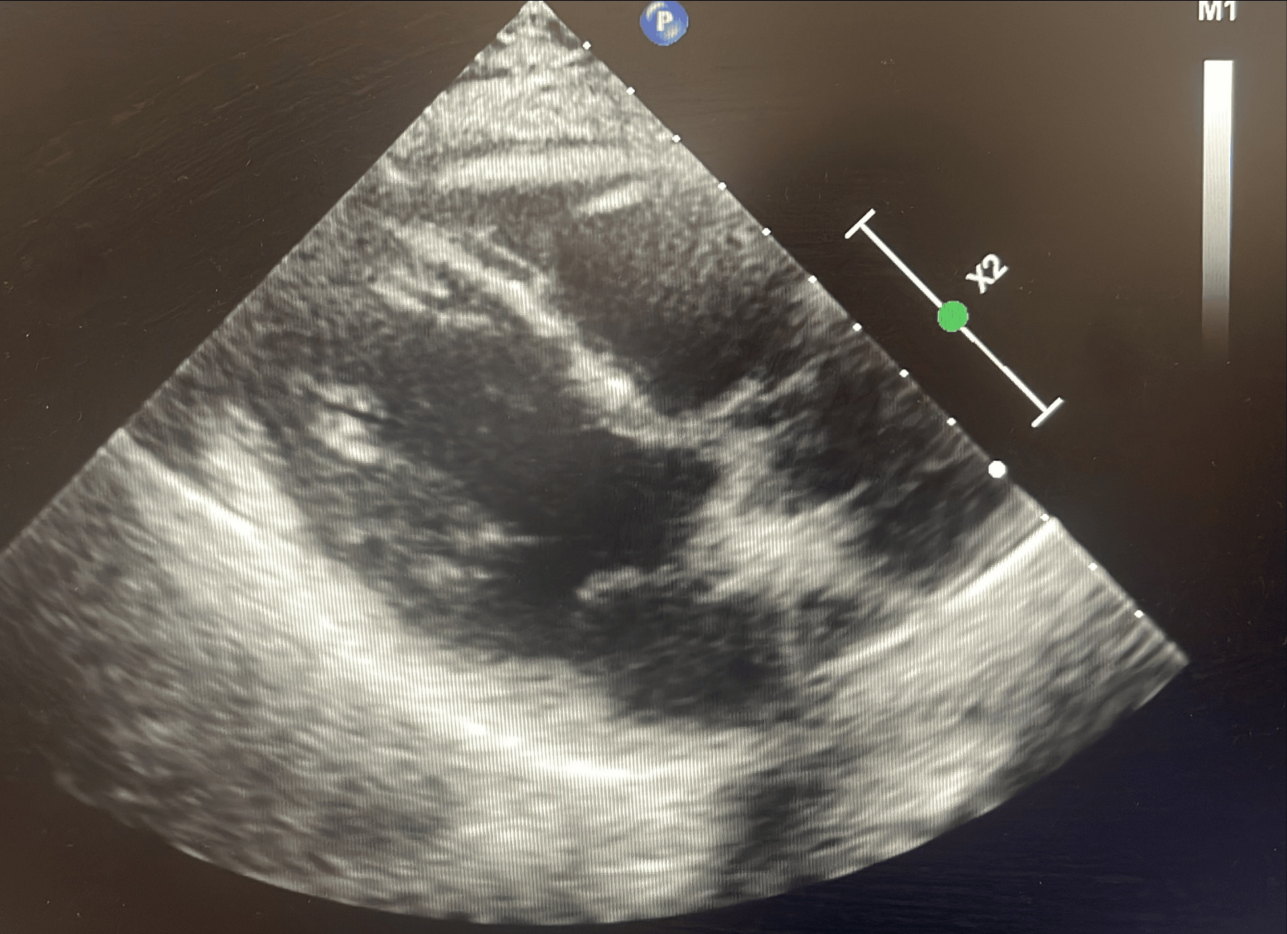
Transthoracic echocardiogram (TTE) showing normal biventricular systolic function with an estimated LVEF of 60–65%. Concentric left ventricular remodelling is observed. No valvular abnormalities or aortic root dilatation are present. The TTE was performed to rule out cardiac involvement, confirming that the disease process was confined to the large vessels.

During admission, the patient reported intermittent flashes of light in her peripheral vision but no visual loss, headaches, or scalp tenderness. Physical examination revealed reduced radial pulses bilaterally and a marked inter-arm blood pressure difference (left arm: 90/69 mmHg; right arm: 137/79 mmHg). Given concern for possible cerebrovascular involvement, particularly transient ischaemic symptoms, a non-contrast CT head was performed, which revealed no evidence of intracranial haemorrhage, infarction, mass lesion, or ischaemic changes, with overall normal intracranial appearances (Figure 3). A transthoracic echocardiogram was requested to assess for aortic root or valvular involvement and to evaluate cardiac function before long-term immunosuppressive therapy. This showed normal biventricular systolic function (LVEF 60-65%), concentric left ventricular remodelling, and no valvular or aortic root abnormalities (Figure 4).

Routine blood tests on admission demonstrated microcytic anaemia (haemoglobin 98 g/L, MCV 76.9 fL) with low serum iron (8 µmol/L) and reduced transferrin saturation (11.2%). C-reactive protein (CRP) was markedly elevated; erythrocyte sedimentation rate (ESR) was not performed. Follow-up testing after treatment showed improvement in haemoglobin (121 g/L), normalisation of serum iron (17 µmol/L) and transferrin saturation (27.9%), and resolution of inflammation (CRP 2 mg/L) (Table 1). Autoantibody and infectious disease screening was notable only for a positive antinuclear antibody (ANA) with a speckled pattern at a titre of 1:160; all other autoantibodies and infectious serologies were negative or within normal limits (Table 2). A chronological summary of the patient’s clinical course, investigations, and management is provided in Table 3.

**Table 2:** Autoantibody and infectious disease screening suggesting nonspecific autoimmune reactivity without active viral infection. ANA: antinuclear antibody; ENA: extractable nuclear antigen; ANCA: anti-neutrophil cytoplasmic antibodies; CCP: cyclic citrullinated peptide; dsDNA: double-stranded DNA; HIV: human immunodeficiency virus; Hep B: hepatitis B; VZV: varicella-zoster virus; IgG: immunoglobulin G

Test Name	Test Result
ANA Pattern	Speckled
ANA Titre	1:160
ENA Screen	Negative
Cytoplasmic ANCA	Negative
Perinuclear ANCA	Negative
Plasma Viscosity	1.90
CCP Antibody	Negative
ANA1	Positive
dsDNA Antibody	Negative
HIV Antibody Screen	Negative
Hep B Surface Antibody	Not detected
Hep B Surface Antigen	Negative
Hepatitis C Antibody Screen	Negative
Hep B Core Antibody	Negative
VZV Immunity IgG	1,317

**Table 3:** Chronological summary of clinical events, investigations, and management. “–” indicates no new relevant findings or management recorded during that period. BP: blood pressure; CRP: C-reactive protein; SMA: superior mesenteric artery; URTI: upper respiratory tract infection; PMR: polymyalgia rheumatica

Timeline from Admission	Events and Symptoms	Key Findings/Investigations	Management/Outcome
Five months before	Diagnosed with declining corticosteroids because of side-effect concerns	-	-
Three months before	Travel to Egypt; self-limiting URTI and chronic diarrhoea (resolved spontaneously)	-	-
One month before	Progressive fatigue, myalgia, reduced exercise tolerance, arm claudication (especially when washing dishes), jaw claudication	-	-
On admission	Constitutional symptoms (20 kg weight loss over three months), no fever, rash, or GI bleed	Blood tests: Hb 98 g/L, MCV 76.9 fL, iron 8 µmol/L, transferrin saturation 11.2%, CRP markedly elevated; ANA positive (1:160 speckled); other autoantibodies negative	Admitted for workup
During admission	Intermittent flashes of light, reduced radial pulses, inter-arm BP difference (L: 90/69, R: 137/79)	CT TAP: diffuse concentric mural thickening of the thoracic and abdominal aorta; complete occlusion L subclavian; stenosis of R subclavian origin; severe stenosis R vertebral artery origin; mild involvement coeliac and SMA; renal arteries normal. CT head: no acute pathology. Echocardiogram: normal systolic function, concentric left ventricular remodelling, no valvular/aortic root abnormality	IV methylprednisolone 500 mg × two doses → oral prednisolone 60 mg daily (tapering). Aspirin, omeprazole, and colchicine. Methotrexate added (later switched to leflunomide)
After discharge	Significant symptomatic improvement	Follow-up bloods: Hb 121 g/L, iron 17 µmol/L, transferrin saturation 27.9%, CRP 2 mg/L	Prednisolone taper ongoing (9 mg/day, reducing by 1 mg/month). Rheumatology follow-up continues

She received two doses of intravenous methylprednisolone 500 mg, followed by oral prednisolone 60 mg daily with a tapering regimen. At her most recent rheumatology review in March 2025, she was maintained on 9 mg prednisolone daily, reducing by 1 mg monthly, alongside aspirin, omeprazole, and colchicine. Methotrexate (20 mg weekly) was commenced for 12 weeks with folic acid supplementation and later switched to leflunomide (20 mg daily). She has shown marked symptomatic improvement and normalisation of inflammatory markers, with ongoing outpatient follow-up.

## Discussion

LVV encompasses disorders such as TA and GCA, which involve chronic inflammation of the aorta and its major branches. The presentation is often non-specific and may include fatigue, weight loss, and anaemia, overlapping with systemic lupus erythematosus (SLE), rheumatoid arthritis (RA), and chronic infections, which can delay diagnosis [1]. In our patient, the combination of significant weight loss, IDA, and prior PMR should have prompted earlier consideration of vasculitis. Targeted questions about GCA symptoms (e.g., scalp tenderness, visual disturbance, jaw claudication) would have been appropriate at the initial presentation. This case highlights the importance of considering LVV even when the presenting features are subtle or mimic other systemic diseases, thereby distinguishing it from many previously reported cases.

PMR and GCA are closely linked, with approximately 50% of GCA patients exhibiting PMR symptoms, and 20% of PMR patients developing GCA [ 8]. Imaging has demonstrated subclinical large-vessel inflammation in up to one-third of isolated PMR cases, most frequently detected by PET/CT (29%), followed by temporal artery biopsy (20%), and ultrasound (15%) [9,10]. In this case, the patient’s PMR diagnosis was followed by the onset of arm and jaw claudication with anaemia, suggesting possible progression to GCA. Temporal artery biopsy, although the gold standard, was not performed because of the strong clinical and imaging evidence and the absence of cranial symptoms, which aligns with current clinical practice.

The patient initially presented with IDA, a common feature of chronic inflammatory disorders. ESR was not performed, but CRP and imaging provided sufficient diagnostic clarity. However, the key diagnostic step was the identification of vascular abnormalities on imaging. Several reports describe LVV presenting with persistent IDA, particularly in younger patients. For example, a 12-year-old girl was treated for IDA without improvement until imaging confirmed TA [11]. Similarly, a 26-year-old woman presented with syncope and dizziness, and magnetic resonance angiography (MRA) revealed thickened walls of the aortic arch and its branches, leading to a diagnosis of TA [12]. These examples highlight that IDA, even in the absence of overt bleeding, can be a presenting clue in LVV and warrants consideration when standard GI and haematologic workups are unrevealing. In this patient, IDA reflected ongoing vascular inflammation, emphasising the pathophysiological link between anaemia and active LVV. Key laboratory values, imaging findings, clinical features, and treatment outcomes in this patient are summarised in Table 4, illustrating the diagnostic reasoning and response to therapy.

**Table 4:** Key laboratory values, imaging findings, clinical features, and treatment outcomes in the patient with LVV, illustrating diagnostic reasoning and response to therapy. CRP: C-reactive protein; MCV: mean corpuscular volume; IDA: iron deficiency anaemia; PMR: polymyalgia rheumatica; TAP: thorax, abdomen, pelvis

Parameter	Findings	Clinical Interpretation and Relevance
Haemoglobin (Hb)	98 g/L at presentation; improved to 121 g/L post-treatment	Reflects IDA and chronic inflammation; recovery indicates response to LVV therapy
MCV	78 fL	Microcytic anaemia consistent with iron deficiency component
CRP	68 mg/L at presentation; normalised post-treatment	Marker of systemic inflammation; elevation supports active vasculitis
Iron Studies	Ferritin low, transferrin saturation decreased	Confirms iron deficiency contributing to anaemia
CT TAP	Diffuse mural thickening of aorta and major branches; multiple vessel occlusions	Key diagnostic modality; revealed extensive LVV involvement
Ultrasound/Doppler	N/A in this patient	Generally useful for assessing vascular inflammation in accessible vessels
Symptoms	Fatigue, weight loss, myalgia, jaw/arm claudication	Correlate with systemic inflammation and ischaemia from vascular involvement
Prior Medical History	PMR	Increases risk for progression to GCA/LVV; supports clinical suspicion
Treatment	High-dose corticosteroids, methotrexate, leflunomide	Led to normalization of labs and symptom improvement; demonstrates effective management

This case is notable for the diagnostic trajectory. Despite typical systemic features of vasculitis, the initial workup focused on anaemia, leading to a CT TAP scan that revealed extensive aortic and branch vessel involvement. The absence of early localised vascular symptoms and prior reluctance to initiate corticosteroid therapy for PMR contributed to a delay in diagnosis. Few cases report LVV being initially suspected through imaging conducted during GI or anaemia workups, highlighting the importance of considering vascular imaging early in patients with unexplained anaemia and systemic symptoms. A clear chronology of symptom onset, diagnostic imaging, and treatment initiation helps demonstrate how clinical reasoning led to the diagnosis.

Imaging is central to both the diagnosis and monitoring of LVV, particularly given the challenges of obtaining tissue biopsies from major vessels. Conventional modalities, including ultrasonography (US), Doppler US, CT angiography (CTA), and MRA, allow evaluation of vascular anatomy and inflammation [3,4,13]. CTA, in particular, is widely accessible, provides excellent spatial resolution, and facilitates a comprehensive assessment of the aorta and its major branches. In this patient, CT thorax, abdomen, and pelvis (TAP) identified diffuse mural thickening and multiple vessel occlusions, directly guiding diagnosis and management. The imaging findings were clinically directed and integral to diagnostic reasoning rather than incidental, reinforcing the need for early vascular imaging in atypical presentations.

High-dose corticosteroids remain the mainstay of LVV treatment, rapidly controlling inflammation and preventing further vascular injury [5,14]. In line with European Alliance of Associations for Rheumatology (EULAR) recommendations, methotrexate and leflunomide were added as steroid-sparing agents [15]. Early initiation of therapy in this patient led to significant clinical improvement, with normalisation of inflammatory markers and recovery of haemoglobin to 121 g/L. The patient’s laboratory and imaging findings, including elevated ESR and CRP, were directly linked to disease activity, and their resolution following therapy further supports the diagnosis and treatment strategy.

## Conclusions

LVV can present atypically with IDA and systemic symptoms such as fatigue and weight loss, often preceding classic vascular signs. In this case, incidental CT imaging revealed extensive aortic and branch involvement, highlighting the importance of early imaging in patients with unexplained anaemia. Prompt recognition and treatment with corticosteroids and immunosuppressive therapy led to significant clinical improvement.
